# Prevalence and correlates of suicidal ideation among the general population in China during the COVID-19 pandemic

**DOI:** 10.1192/j.eurpsy.2021.5

**Published:** 2021-02-03

**Authors:** Le Shi, Jian-Yu Que, Zheng-An Lu, Yi-Miao Gong, Lin Liu, Yun-He Wang, Mao-Sheng Ran, Nisha Ravindran, Arun V. Ravindran, Seena Fazel, Yan-Ping Bao, Jie Shi, Lin Lu

**Affiliations:** 1 Peking University Sixth Hospital, Peking University Institute of Mental Health, NHC Key Laboratory of Mental Health (Peking University), National Clinical Research Center for Mental Disorders (Peking University Sixth Hospital), Peking University, Beijing, China; 2 Peking-Tsinghua Center for Life Sciences and PKU-IDG/McGovern Institute for Brain Research, Peking University, Beijing, China; 3 National Institute on Drug Dependence and Beijing Key Laboratory on Drug Dependence Research, Peking University, Beijing, China; 4 Department of Social Work and Social Administration, University of Hong Kong, Hong Kong, China; 5 Department of Psychiatry, University of Toronto, Toronto, Canada; 6 Department of Psychiatry, University of Oxford, Oxford, United Kingdom

**Keywords:** China, COVID-19, prevalence, risk factors, suicidal ideation

## Abstract

**Background:**

The coronavirus disease 2019 (COVID-19) pandemic is a major threat to the public. However, the comprehensive profile of suicidal ideation among the general population has not been systematically investigated in a large sample in the age of COVID-19.

**Methods:**

A national online cross-sectional survey was conducted between February 28, 2020 and March 11, 2020 in a representative sample of Chinese adults aged 18 years and older. Suicidal ideation was assessed using item 9 of the Patient Health Questionnaire-9. The prevalence of suicidal ideation and its risk factors was evaluated.

**Results:**

A total of 56,679 participants (27,149 males and 29,530 females) were included. The overall prevalence of suicidal ideation was 16.4%, including 10.9% seldom, 4.1% often, and 1.4% always suicidal ideation. The prevalence of suicidal ideation was higher in males (19.1%) and individuals aged 18–24 years (24.7%) than in females (14.0%) and those aged 45 years and older (11.9%). Suicidal ideation was more prevalent in individuals with suspected or confirmed infection (63.0%), frontline workers (19.2%), and people with pre-existing mental disorders (41.6%). Experience of quarantine, unemployed, and increased psychological stress during the pandemic were associated with an increased risk of suicidal ideation and its severity. However, paying more attention to and gaining a better understanding of COVID-19-related knowledge, especially information about psychological interventions, could reduce the risk.

**Conclusions:**

The estimated prevalence of suicidal ideation among the general population in China during COVID-19 was significant. The findings will be important for improving suicide prevention strategies during COVID-19.

## Introduction

Suicide is a significant global public health problem that occurs across lifespans. According to the World Health Organization, one person dies of suicide every 40 s [1]. In addition to completed suicide, suicidal behaviors, especially suicidal ideation, are increasingly common. The estimated lifetime prevalence of suicidal ideation in the general population is approximately 4.0% [2,3]. Reports state that 13.2% of those reporting suicidal ideation during the past year have later attempted suicide in the past 12 months [4]. Improving the understanding of the characteristics and correlates of suicidal ideation is essential to inform suicide prevention and provide intervention targets.

Suicidal behaviors are multifactorial and involve complex interactions between biological and environmental determinants [5]. Among them, one key risk factor in the etiology of suicidal ideation is an adverse life stressor [6]. Numerous studies have proved the critical role of multiple life events, such as adverse early life experiences, unemployment, and social isolation, in suicidal behaviors [7]. This may be because environmental changes could induce psychological stress [8], and lead to neuropsychological changes that increase suicidal behaviors [9]. Moreover, there is a dose–response relationship between psychological stress and suicide possibility [10], suggesting that severe stressful events may significantly increase the risk of suicidal behaviors.

The epidemic of communicable disease, as a major life stressor in both cases and surrounding people, can have profound impacts on mental health [11]. However, few studies have focused on the relationship between suicidal behaviors and infectious disease outbreaks. Evidence showed that approximately 10–20% of Ebola survivors had thoughts of suicide or self-harm [12]. During the severe acute respiratory syndrome (SARS) epidemic, a high rate of suicide deaths was reported in the elderly based on court records [13,14]. These findings from other infectious disease outbreaks confirm the need for the early identification of vulnerable populations. Developing a comprehensive profile of suicidal ideation is the key first step in this direction.

In December 2019, coronavirus disease 2019 (COVID-19) characterized by high contagiousness and rapid transmission occurred [15]. The World Health Organization declared COVID-19 a pandemic in March 2020 [16]. As of December 27, 2020, more than 79 million people were infected and 1 million confirmed cases died across 222 countries, areas, or territories [17]. Without available vaccines and specific antiviral drugs, some restrictive interventions, such as quarantine, social distancing, and work at home, were used to control the viral spread [18,19]. The unpredictability and uncertainty of the COVID-19 pandemic, fear of infection, and COVID-19-related environmental changes are major stressors that trigger a series of mental health problems [20,21]. Reports since the onset of the pandemic have documented psychological symptom profiles [22,23]. As a major rigorous crisis [24], suicidal ideation and associated factors in the general population should be systematically investigated. Preliminary reports of suicide surges were reported in several studies during the early phase of the pandemic [25,26], and some factors, such as socioeconomic disadvantage, low social support, increased burden, and unemployment, might be associated with suicidal behaviors [27-29]. However, to date, the comprehensive evaluation of suicidal ideation during the COVID-19 pandemic is still lacking.

The objectives of this study with a large nationwide sample were to explore the prevalence of suicidal ideation and its risk factors among the general population in China during the COVID-19 pandemic, and further provide evidence for suicide prevention under a public health emergency.

## Methods

### Study design

An anonymous national cross-sectional online survey with convenience sampling was conducted from February 28, 2020 to March 11, 2020, during which time the cumulative cases of COVID-19 reached a peak in China. Users aged over 18 years of the Chinese website Joybuy, a large e-commerce and information service company that provides online health products and services, were invited to participate in this self-administered survey voluntarily until the convenience sample covered all 34 provincial-level regions in China. The detailed procedure was described in our previous publication [22]. In brief, 71,227 individuals clicked on the survey page. Of that number, 56,932 participants completed the survey, with a response rate of 79.9%. After data quality control evaluation, 56,679 participants from 34 province-level regions in China were finally included in the analyses, with an effective rate of 99.6%. The authors assert that all procedures contributing to this work comply with the ethical standards of the relevant national and institutional committees on human experimentation and with the Helsinki Declaration of 1975, as revised in 2008, and were approved by the Peking University Sixth Hospital Institutional Review Board.

### Measurements

Information on demographic characteristics, COVID-19-related factors, psychological health literacy, and suicidal ideation were collected in this survey. Demographic characteristics included age, gender, location, marital status, educational level, and monthly family income. Questions pertaining to past history and family history of mental disorders, and history of sleep problems were also asked. The survey questions included inquiries into COVID-19 specific factors that may contribute to suicidal ideation, whether infected with COVID-19 or not, exposed to the most severely affected region recently, experiences of quarantine and unemployment, and participation in frontline work. Frontline work referred to work that had direct contact with confirmed cases of COVID-19 [22]. In addition, attention to COVID-19-related information and perceived knowledge of COVID-19 was assessed using a visual analog scale, where zero indicates paying no attention or ignorant of COVID-19-related information, while 10 indicates being highly knowledgeable and paying constant attention to COVID-19-related information.

The psychological information were evaluated in three domains: (a) perceived psychological stress before and after the COVID-19 pandemic using a visual analog scale, (b) seeking psychological knowledge following the COVID-19 outbreak, and (c) degree of access to information about psychological interventions during the pandemic. Item 9 of the Patient Health Questionnaire-9 was used to assess the presence of suicidal ideation as follows: “Over the last two weeks, how often have you been bothered by thoughts that you would be better off dead, or of hurting yourself in some way?” The response options were “not at all,” “several days,” “more than half the days,” and “nearly every day.” Suicidal ideation was defined as positive if respondents answered, “several days,” “more than half the days,” and “nearly every day,” or negative if answered “not at all” [30,31]. In addition, according to the various degrees of suicidal ideation, the participants with suicidal ideation were further divided into three groups: seldom (several days), often (more than half the days), and always (nearly every day) suicidal ideation.

### Data analysis

Descriptive statistics were used to present demographic, COVID-19-related, and psychological data. Means with standard deviations were used to describe continuous variables, and numbers with percentages were used to describe categorical variables. The prevalence with 95% confidence interval (CI) of suicidal ideation in different demographic characteristics and COVID-19-related factors was reported by gender. Tests of *χ* ^2^ were used to assess statistical differences in categorical variables. The associations between suicidal ideation and relevant covariates were analyzed using the multivariable logistic regression model, and factors associated with the severity of suicidal ideation were examined using ordinal logistic regression analysis. In addition, the participants were divided into three groups based on their changes in psychological stress after the COVID-19 pandemic, and potential factors associated with suicidal ideation in each group were explored using multivariable logistic regression analysis. Multivariable adjusted odds ratios (ORs) with 95% CIs were reported. Data analyses were conducted using the SPSS 20 software. All tests were two-sided, with *p*-value less than 0.05, considered significant.

## Results

### Descriptive characteristics

In this national cross-sectional online survey, 56,679 participants (27,149 [47.9%] males and 29,530 [52.1%] females) from 34 province-level regions were included in the final analysis. Table 1 presents the characteristics of 47,357 (83.6%) participants without suicidal ideation and 9,322 (16.4%) participants with suicidal ideation. Of these, 6,206 (10.9%) participants seldom reported suicidal ideation, 2,320 (4.1%) often had suicidal ideation, and 796 (1.4%) always reported suicidal ideation over the past 2 weeks (see Supplementary Table 1). Most participants with suicidal ideation were male (55.7%), aged 18–44 years (88.9%), living in urban areas (91.8%), married (72.9%), and had an educational level of college and higher (80.9%). In addition, 63 (0.7%) of them were suspected or confirmed to be infected with COVID-19 and 1,863 (20.0%) participated in frontline work. A total of 3,202 (34.3%) respondents with suicidal ideation experienced quarantine, and 666 (7.1%) reported unemployment. Also, 1,531 (16.4%) of the 9,322 participants with suicidal ideation had more access to psychological information during the COVID-19 pandemic.

**Table 1.** Demographic characteristics of participants.

	Total	Suicidal ideation	No suicidal ideation
Overall, *n* (%)	56,679(100)	9,322(16.4)	47,357(83.6)
Categorical variables, *n* (%)			
Gender, *n* (%)			
Male	27,149(47.9)	5,195(55.7)	21,954(46.4)
Female	29,530(52.1)	4,127(44.3)	25,403(53.6)
Age (years), *n* (%)			
18–24	3,267(5.8)	806(8.6)	2,461(5.2)
25–34	23,050(40.7)	4,303(46.2)	18,747(39.6)
35–44	21,658(38.2)	3,181(34.1)	18,477(39.0)
≥45	8,704(15.4)	1,032(11.1)	7,672(16.2)
Living area, *n* (%)			
Urban	52,839(93.2)	8,562(91.8)	44,277(93.5)
Rural	3,840(6.8)	760(8.2)	3,080(6.5)
Geographical region in China, *n* (%)			
Eastern	23,172(40.9)	3,731(40.1)	19,441(41.1)
Northern	10,227(18.0)	1,605(17.2)	8,622(18.2)
Northwest	1,348(2.4)	256(2.7)	1,092(2.3)
Northeast	3,921(6.9)	687(7.4)	3,234(6.8)
Central	4,803(8.5)	858(9.2)	3,945(8.3)
Southern	10,028(17.7)	1,638(17.6)	8,390(17.7)
Southwest	3,156(5.6)	537(5.8)	2,619(5.5)
Missing values	24(0.0)		
Marital status, *n* (%)			
Married	43,763(77.2)	6,799(72.9)	36,964(78.1)
Separated/divorced	1,270(2.2)	194(2.1)	1,076(2.3)
Never married	11,646(20.5)	2,329(25.0)	9,317(19.7)
Educational level, *n* (%)			
Middle school and lower	9,540(16.8)	1,779(19.1)	7,761(16.4)
College and higher	47,139(83.2)	7,543(80.9)	39,596(83.6)
Monthly family income (¥), *n* (%)			
<5,000	13,016(23.0)	2,752(29.5)	10,264(21.7)
5,000–7,999	13,663(24.1)	2,433(26.1)	11,230(23.7)
8,000–11,999	12,829(22.6)	1,978(21.2)	10,851(22.9)
≥12,000	17,171(30.3)	2,159(23.2)	15,012(31.7)
History of mental disorders, *n* (%)			
No	56,137(99.0)	9,097(97.6)	47,040(99.3)
Unknown	381(0.7)	158(1.7)	223(0.5)
Yes	161(0.3)	67(0.7)	94(0.2)
Family history of mental disorders, *n* (%)			
No	55,675(98.2)	9,047(97.0)	46,628(98.5)
Unknown	608(1.1)	198(2.1)	410(0.9)
Yes	396(0.7)	77(0.8)	319(0.7)
History of sleep problems, *n* (%)			
No	40,698(71.8)	5,873(63.0)	34,825(73.5)
Yes	15,981(28.2)	3,449(37.0)	12,532(26.5)
Have you suspected or confirmed been infected during the COVID-19 pandemic? *n* (%)			
No	56,579(99.8)	9,259(99.3)	47,320(99.9)
Yes	100(0.2)	63(0.7)	37(0.1)
Have you been to the most severely COVID-19 affected region recently? *n* (%)			
No	53,982(95.2)	8,708(93.4)	45,274(95.6)
Yes, I remain at the region	2,352(4.1)	531(5.7)	1,821(3.8)
Yes, I have left the region	345(0.6)	83(0.9)	262(0.6)
Have you experienced quarantine during the COVID-19 pandemic? *n* (%)			
No	40,225(71.0)	6,120(65.7)	34,105(72.0)
Yes, quarantine duration < 14 days	2,199(3.9)	531(5.7)	1,668(3.5)
Yes, quarantine duration ≥ 14 days	14,255(25.2)	2,671(28.7)	11,584(24.5)
Have you experienced the COVID-19 induced unemployment? *n* (%)			
No	54,589(96.3)	8,656(92.9)	45,933(97.0)
Yes	2,090(3.7)	666(7.1)	1,424(3.0)
Did you participate in frontline work? *n* (%)			
No	46,954(82.8)	7,459(80.0)	39,495(83.4)
Yes	9,725(17.2)	1,863(20.0)	7,862(16.6)
Have you got more access to psychological knowledge after the COVID-19 pandemic? *n* (%)			
No	45,613(80.5)	7,791(83.6)	37,822(79.9)
Yes	11,066(19.5)	1,531(16.4)	9,535(20.1)
Continuous variables			
The rating of perceived psychological stress after the COVID-19 pandemic (mean [SD])	4.27 ± 2.70	5.31 ± 2.53	4.06 ± 2.68
The degree of attention to the related information about the COVID-19 pandemic (mean [SD])	9.21 ± 1.65	8.84 ± 2.09	9.29 ± 1.54
The mastery degree of perceived knowledge of COVID-19 (mean [SD])	8.11 ± 1.87	7.73 ± 2.17	8.19 ± 1.80
The difficulty in getting access to the information about psychological interventions after the COVID-19 pandemic (mean [SD])	3.26 ± 2.52	4.50 ± 2.44	3.02 ± 2.46

Note: COVID-19, coronavirus disease 2019; SD, standard deviation.

### Prevalence of suicidal ideation stratified by sampling clusters

**Figure 1.**
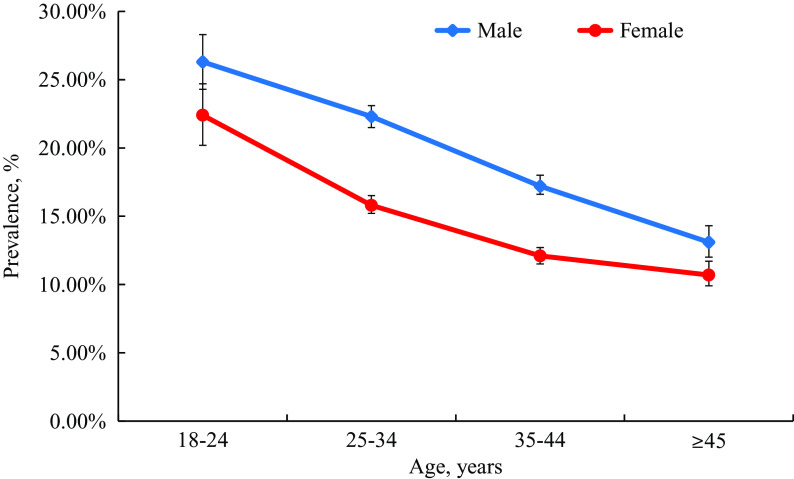
Prevalence of suicidal ideation in different gender with different ages during the COVID-19 pandemic. Error bars represent 95% CI.

The overall prevalence of suicidal ideation was 16.4% (95% CI, 16.2–16.8) among the population surveyed during the pandemic. Males had a higher prevalence of various degrees of suicidal ideation (19.1%; 95% CI, 18.7–19.6) than females (14.0%; 95% CI, 13.6–14.4; *p* < 0.001), and the prevalence significantly decreased from the 18 to 24 years group (male: 26.3%; 95% CI, 24.3–28.3. female: 22.4%; 95% CI, 20.2–24.7) compared to those aged 45 years or older (male: 13.1%; 95% CI, 12.1–14.2. female: 10.7%; 95% CI, 9.9–11.7; Figure 1). As shown in Table 2, the prevalence of suicidal ideation decreased significantly with increasing monthly family income, from 21.1% (95% CI, 20.4–21.9) in participants with less than 5,000 RMB per month to 12.6% (95% CI, 12.1–13.1; *p* < 0.001) in participants with 12,000 RMB or higher monthly family income. Furthermore, people from areas with relatively low-income levels (rural areas: 19.8%; 95% CI, 18.6–21.1. northwest regions: 19.0%; 95% CI, 17.0–21.2) reported a higher prevalence of suicidal ideation compared to those from high economic development areas. Individuals with a history of mental disorders (41.6%; 95% CI, 34.0–49.6) and sleep problems (21.6%; 95% CI, 21.0–22.2) had a higher prevalence of suicidal ideation than those without a history of mental disorders (16.2%; 95% CI, 15.9–16.5; *p* < 0.001) or sleep problems (14.4%; 95% CI, 14.1–14.8; *p* < 0.001). During the COVID-19 pandemic, individuals with confirmed or suspected infection by COVID-19 (63.0%; 95% CI, 52.7–72.3) reported higher rates of suicidal ideation compared to uninfected individuals (16.4%; 95% CI, 16.1–16.7; *p* < 0.001). Participants who were quarantined for more than 14 days (18.7%; 95% CI, 18.1–19.4) had a significantly higher prevalence of suicidal ideation than those without quarantine (15.2%; 95% CI, 14.9–15.6; *p* < 0.001). Surprisingly, the prevalence of suicidal ideation was found to be higher in those who were partially quarantined than in those who completed it. Individuals with unemployment also showed increased suicidal ideation (31.9%; 95% CI, 29.9–33.9) compared to those without experience of unemployment (15.9%; 95% CI, 15.6–16.2; *p* < 0.001). The prevalence of suicidal ideation in frontline workers (19.2%; 95% CI, 18.4–20.0) was significantly higher than those who were not in the frontline (15.9%; 95% CI, 15.6–16.2; *p* < 0.001). However, individuals who had more access to psychological information after the COVID-19 outbreak (13.8%; 95% CI, 13.2–14.5) had a lower rate of suicidal ideation than those with less access (17.1%; 95% CI, 16.7–17.4; *p* < 0.001).

**Table 2.** Prevalence (%) of suicidal ideation stratified by sampling clusters during the COVID-19 pandemic.

	Male (%) (95% CI)	Female (%) (95% CI)	Total (%) (95% CI)
Overall	19.1(18.7–19.6)	14.0(13.6–14.4)	16.4(16.2–16.8)
Age (years)			
18–24	26.3(24.3–28.3)	22.4(20.2–24.7)	24.7(23.2–26.2)
25–34	22.3(21.5–23.1)	15.8(15.2–16.5)	18.7(18.2–19.2)
35–44	17.2(16.6–18.0)	12.1(11.5–12.7)	14.7(14.2–15.2)
≥45	13.1(12.1–14.2)	10.7(9.9–11.7)	11.9(11.2–12.6)
*p*	<0.001	<0.001	<0.001
Living area			
Urban	18.9(18.4–19.4)	13.7(13.4–14.2)	16.2(15.9–16.5)
Rural	22.9(21.0–24.9)	17.1(15.5–18.8)	19.8(18.6–21.1)
*P*	<0.001	<0.001	<0.001
Geographical region in China			
Eastern	18.9(18.2–19.6)	13.5(12.9–14.2)	16.1(15.6–16.6)
Northern	18.8(17.7–20.0)	13.2(12.3–14.1)	15.7(15.0–16.4)
Northwest	19.8(18.0–21.8)	16.3(13.4–19.9)	19.0(17.0–21.2)
Northeast	20.7(18.0–23.7)	15.7(14.3–17.3)	17.5(16.4–18.8)
Central	20.7(19.2–22.4)	14.4(13.0–15.9)	17.9(16.8–19.0)
Southern	14.3(13.4–15.3)	14.3(13.4–15.3)	16.3(15.6–17.1)
Southwest	18.6(16.8–20.5)	15.2(13.4–17.2)	17.0(15.7–18.4)
*p*	0.251	0.015	0.001
Marital status			
Married	18.1(17.5–18.6)	13.4(13.0–13.8)	15.5(15.2–15.9)
Separated/divorced	18.1(14.9–21.8)	13.5(11.2–16.1)	15.3(13.4–17.4)
Never married	22.4(21.4–23.4)	16.8(15.8–17.9)	20.0(19.3–20.7)
*p*	<0.001	<0.001	<0.001
Educational level			
Middle school and lower	21.9(20.6–23.2)	16.3(15.3–17.3)	18.6(17.9–19.5)
College and higher	18.7(18.2–19.2)	13.5(13.0–13.9)	16.0(15.7–16.3)
*p*	<0.001	<0.001	<0.001
Monthly family income (¥)			
<5,000	24.9(23.8–26.1)	18.3(17.5–19.2)	21.1(20.4–21.9)
5,000–7,999	20.8(19.8–21.8)	15.0(14.2–15.9)	17.8(17.2–18.5)
8,000–11,999	18.3(17.4–19.3)	12.6(11.8–13.5)	15.4(14.8–16.1)
≥12,000	14.8(14.0–15.5)	10.3(9.7–11.0)	12.6(12.1–13.1)
*p*	<0.001	<0.001	<0.001
History of mental disorders			
No	18.8(18.4–19.3)	13.8(13.4–14.2)	16.2(15.9–16.5)
Unknown	45.0(38.4–51.8)	36.6(29.3–44.6)	41.5(36.5–46.6)
Yes	51.1(40.4–61.7)	29.6(19.6–41.8)	41.6(34.0–49.6)
*p*	<0.001	<0.001	<0.001
Family history of mental disorders			
No	18.9(18.5–19.4)	13.8(13.4–14.2)	16.2(16.0–16.6)
Unknown	35.9(30.7–41.4)	28.8(23.7–34.5)	32.6(28.9–36.5)
Yes	21.1(15.8–27.6)	17.8(12.8–24.0)	19.4(15.7–23.8)
*p*	<0.001	<0.001	<0.001
History of sleep problems			
No	16.8(16.3–17.4)	12.2(11.7–12.6)	14.4(14.1–14.8)
Yes	25.3(24.3–26.3)	18.4(17.6–19.2)	21.6(21.0–22.2)
*p*	<0.001	<0.001	<0.001
Have you suspected or confirmed been infected during the COVID-19 pandemic?			
No	19.0(18.6–19.5)	13.9(13.5–14.3)	16.4(16.1–16.7)
Yes	66.7(54.2–77.3)	54.8(36.3–72.2)	63.0(52.7–72.3)
*p*	<0.001	<0.001	<0.001
Have you been to the most severely COVID-19 affected region recently?			
No	18.7(18.2–19.2)	13.8(13.4–14.2)	16.1(15.8–16.4)
Yes, I remain at the region	26.7(24.3–29.2)	17.8(15.6–20.2)	22.6(20.9–24.3)
Yes, I have left the region	32.0(25.2–39.6)	16.2(11.2–22.7)	24.1(19.7–29.0)
*p*	<0.001	0.001	<0.001
Have you ever experienced quarantine during the COVID-19 pandemic?			
No	17.8(17.2–18.3)	12.9(12.5–13.4)	15.2(14.9–15.6)
Yes, quarantine duration <14 days	27.3(24.7–30.0)	20.8(18.4–23.4)	24.1(22.4–26.0)
Yes, quarantine duration ≥14 days	21.6(20.7–22.6)	16.1(15.2–16.9)	18.7(18.1–19.4)
*p*	<0.001	<0.001	<0.001
Have you experienced the COVID-19 induced unemployment?			
No	18.5(18.0–19.0)	13.4(13.0–13.8)	15.9(15.6–16.2)
Yes	35.7(32.7–38.8)	28.5(25.8–31.2)	31.9(29.9–33.9)
*p*	<0.001	<0.001	<0.001
Did you participate in frontline work?			
No	18.6(18.0–19.1)	13.7(13.3–14.1)	15.9(15.6–16.2)
Yes	21.3(20.2–22.4)	16.0(14.9–17.2)	19.2(18.4–20.0)
*p*	<0.001	<0.001	<0.001
Have you got more access to psychological knowledge after the COVID-19 pandemic?			
No	19.7(19.2–20.3)	14.5(14.1–15.0)	17.1(16.7–17.4)
Yes	16.3(15.3–17.4)	12.0(11.2–12.9)	13.8(13.2–14.5)
*p*	<0.001	<0.001	<0.001

### Factors associated with suicidal ideation during the COVID-19 pandemic

Table 3 shows the results of the multivariable-adjusted logistic regression model. Male gender, younger age, unmarried status, lower family income, history of mental disorders and sleep problems, and family history of mental disorders were associated with an increased risk of suicidal ideation. Psychological and COVID-19-related factors, including confirmed or suspected infection with COVID-19 (OR, 3.50; 95% CI, 2.20–5.59; *p* < 0.001), living in the most severely affected region (still remaining: OR, 1.47; 95% CI, 1.29–1.67; *p* < 0.001. left: OR, 1.54; 95% CI, 1.17–2.02; *p* = 0.002), experience of quarantine (duration < 14 days: OR, 1.39; 95% CI, 1.24–1.55; *p* < 0.001. duration ≥ 14 days: OR, 1.11; 95% CI, 1.05–1.17; *p* < 0.001), unemployment (OR, 1.77; 95% CI, 1.60–1.97; *p* < 0.001), participating in frontline works (OR, 1.21; 95% CI, 1.14–1.29; *p* < 0.001), higher perceived psychological stress during the COVID-19 pandemic (OR, 1.12; 95% CI, 1.11–1.13; *p* < 0.001), and more difficulty in access to information about psychological interventions (OR, 1.19; 95% CI, 1.17–1.20; *p* < 0.001) were significantly associated with the increased risk of suicidal ideation, whereas paying more attention to information about COVID-19 pandemic (OR, 0.88; 95% CI, 0.87–0.89; *p* < 0.001), better understanding of COVID-19-related knowledge (OR, 0.95; 95% CI, 0.94–0.97; *p* < 0.001), and getting more access to knowledge about psychological interventions during the COVID-19 pandemic (OR, 0.74; 95% CI, 0.69–0.79; *p* < 0.001) were associated with lower odds of suicidal ideation.

**Table 3.** Multivariable analysis of factors associated with suicidal ideation during the COVID-19 pandemic.

Variables	OR (95% CI)	*p*
Gender		
Male	1(ref)	
Female	0.65(0.62–0.69)	<0.001
Age (years)		
18–24	1.94 (1.72–2.20)	<0.001
25–34	1.57(1.45–1.70)	<0.001
35–44	1.24(1.14–1.34)	<0.001
≥45	1(ref)	
Living area		
Urban	1(ref)	
Rural	1.08(0.98–1.18)	0.109
Geographical region in China		
Eastern	1(ref)	
Northern	0.94(0.88–1.01)	0.083
Northwest	0.96(0.83–1.12)	0.638
Northeast	1.06(0.96–1.16)	0.260
Central	0.77(0.70–0.86)	<0.001
Southern	0.83(0.78–0.89)	<0.001
Southwest	0.91(0.82–1.01)	0.087
Marital status		
Married	1(ref)	
Separated/divorced	0.98(0.83–1.16)	0.798
Never married	0.92(0.86–0.98)	0.013
Educational level		
Middle school and lower	1(ref)	
College and higher	0.94(0.88–1.01)	0.083
Monthly family income (¥)		
<5,000	1(ref)	
5,000–7,999	0.86(0.80–0.92)	<0.001
8,000–11,999	0.76(0.71–0.81)	<0.001
≥12,000	0.62(0.58–0.67)	<0.001
History of mental disorders		
No	1(ref)	
Unknown	1.96(1.50–2.57)	<0.001
Yes	2.13(1.47–3.08)	<0.001
Family history of mental disorders		
No	1(ref)	
Unknown	1.16(0.92–1.45)	0.210
Yes	0.68(0.51–0.90)	0.008
History of sleep problems		
No	1(ref)	
Yes	1.45(1.38–1.53)	<0.001
Have you suspected or confirmed been infected during the COVID-19 pandemic?		
No	1(ref)	
Yes	3.50(2.20–5.59)	<0.001
Have you been to the most severely COVID-19 affected region recently?		
No	1(ref)	
Yes, I remain at the region	1.47(1.29–1.67)	<0.001
Yes, I have left the region	1.54(1.17–2.02)	0.002
Have you ever experienced quarantine during the COVID-19 pandemic?		
No	1(ref)	
Yes, quarantine duration <14 days	1.39(1.24–1.55)	<0.001
Yes, quarantine duration ≥14 days	1.11(1.05–1.17)	<0.001
Have you experienced the COVID-19 induced unemployment?		
No	1(ref)	
Yes	1.77(1.60–1.97)	<0.001
Did you participate in frontline work?		
No	1(ref)	
Yes	1.21(1.14–1.29)	<0.001
Have you got more access to psychological knowledge after the COVID-19 pandemic?		
No	1(ref)	
Yes	0.74(0.69–0.79)	<0.001
The rating of perceived psychological stress after the COVID-19 pandemic	1.12(1.11–1.13)	<0.001
The degree of attention to the related information about COVID-19 pandemic	0.88(0.87–0.89)	<0.001
The mastery degree of perceived knowledge of COVID-19	0.95(0.94–0.97)	<0.001
The difficulty in getting access to the information about psychological interventions after the COVID-19 pandemic	1.19(1.17–1.20)	<0.001
Nagelkerke *R* ^2^	0.150	

The ordinal logistic regression analysis identified several demographic and clinical characteristics and COVID-19-related factors that were associated with the various degrees of suicidal ideation, which were consistent with the variables related to the overall suicidal ideation (see Supplementary Table 2). In addition, some factors (e.g., quarantine, unemployment, frontline work, and access to information of the COVID-19 pandemic and psychological knowledge) were independently associated with the risk of suicidal ideation across different groups of distinct changes in psychological stress (see Supplementary Table 3), further validating the relationship of COVID-19 experiences and suicidal ideation.

## Discussion

This study of a large nationwide sample from China found a relatively high prevalence of suicidal ideation among the general population during the COVID-19 pandemic. Several factors have been shown to be independently associated with increased risk for suicidal ideation and its various severities, including basic demographic and clinical characteristics (gender, age, marital status, family income level, and history of mental disorders and sleep problems), epidemic-related experience (history of infection, ever been to the most severely affected region, quarantine, unemployment, participation in frontline work, and COVID-19-related information exposure), and current psychological state (perceived psychological stress and access to information on psychological services). These findings provide a comprehensive profile of suicidal ideation in the general population during the COVID-19 pandemic, and suggest the importance of relevant mental health policy-making and suicide prevention in a public health emergency.

Compared to previous infectious disease outbreaks, such as SARS and Middle East respiratory syndrome, COVID-19 is associated with faster contagion and affects more people [32]. Fear of contagion, shortage of protective equipment, high-intensity rescue work, social isolation, and a series of environmental changes may lead to suicidal behaviors [24]. Consistent with previous reports [33-36], this study found that people at risk, such as those with confirmed and suspected infection, frontline workers, and people with pre-existing mental disorders, presented a high prevalence of suicidal ideation. In addition, those with the most severe COVID-19 affected region showed increased suicidal ideation, which may be related to psychological problems due to a higher risk of exposure to COVID-19 [22]. Our prior studies have noted that frontline workers, especially medical staff, during the COVID-19 pandemic were vulnerable to psychological problems [22,37], and frontline healthcare workers in Wuhan, the city most affected by COVID-19 in China, showed the greatest severity of distress symptoms [38]. Considering the high likelihood of a prolonged COVID-19 pandemic, more preventive strategies for suicidal behaviors should be delivered to these susceptible populations.

The results of this study showed that young adults and males are more vulnerable to experiencing various degrees of suicidal ideation compared to older populations and females. This is consistent with our previous findings that young people are more prone to depression, anxiety, insomnia, and acute stress, and males were vulnerable to depression, insomnia, and acute stress during the COVID-19 pandemic [22]. It has been proposed that lack of physical activity increases the likelihood of suicidal behaviors in young people [39]. Regarding gender, previous studies have indicated that females have higher suicidal ideation than males [40,41]. However, higher rates of COVID-19 infection in males may have contributed to the greater prevalence found in our study [42]. It follows that young men with a higher prevalence of suicidal ideation should be a priority group for targeted suicide prevention.

In this survey, the experience of quarantine and unemployment was found to be associated with suicidal ideation across populations with different changes in psychological stress during the pandemic. To mitigate the rapid transmission of COVID-19 [43,44], quarantine was employed by most countries, including China. However, quarantine has a significant negative impact on mental wellness and is well documented to be associated with depression, anxiety, and increased stress because of fear of infection, social isolation, insufficient supply, inadequate information, financial loss, and stigma [45]. Some cases of suicidal behaviors associated with quarantine have been reported since the outbreak of COVID-19 [46,47]. Moreover, this study found that those who underwent partial quarantine experience (less than 14 days) had a higher risk of suicidal ideation than those with a long quarantine duration. This may be because sufficient quarantine duration may decrease the possibility of COVID-19 infection, and reduce the risk of viral spread. Combined with previous and current findings, quarantine with sufficient time (e.g., over 14 days) and social connection are needed to prevent from suicidal behaviors during the COVID-19 pandemic [48].

In addition to quarantine, unemployment was also found in this study to increase the risk of suicidal ideation. Published suicide literature unrelated to pandemics has confirmed a direct relationship between unemployment and suicidal ideation [49], such that for every percentage increase in unemployment, the suicide rate increases by approximately 0.6% [50]. At the initial stage of the COVID-19 pandemic, the effects on unemployment were evaluated [51]. In this study, 3.7% of participants had experience of unemployment, and of this one third or more reported suicidal ideation. Moreover, in contrast to people with high-family income and from economically developed areas, we observed relatively higher reports of suicidal ideation in the low family income and economically undeveloped areas group. These findings suggest that during the infectious disease pandemic, economic, and psychological support is necessary for people experiencing unemployment and financial problems.

Attention to and knowledge of COVID-19-related information is another important factor in suicidal ideation. People who were more attentive to information on the pandemic and had a better understanding of its consequences had a lower prevalence of suicidal ideation. However, other researchers have reported a positive link between information exposure to COVID-19 and emotional distress [52]. This discrepancy may be due to the source of the information and may also be influenced by personal COVID-19-related experiences [53]. For example, COVID-19 information from medical staff might cause less psychological distress compared to that from the Internet, and healthcare workers with formal lessons could cope better with psychological problems [54]. Specifically, having reliable knowledge about psychological interventions was a protective factor against the severity of suicidal ideation during the COVID-19 pandemic in this study. Effective education as part of mental health care will likely alleviate the adverse psychological impact and reduce the likelihood of suicide [55,56]. Digital and social media are more suitable for disseminating mental health knowledge than traditional healthcare, which has a deficiency of limited access, especially in lower and middle-income countries [57,58]. Furthermore, digital technology and social media can greatly enhance the quick dissemination of time-sensitive health information in pandemics and is particularly useful to reach young adults who were proven to be vulnerable populations of suicidal ideation in this study. In future, these dissemination channels should be systematically evaluated for effectiveness and sustainability.

This study, with a relatively large sample, systematically researched the profile of suicidal ideation in China during the COVID-19 pandemic. However, this study had several limitations. First, it was a cross-sectional study, and the causal relationship between multiple risk factors, such as quarantine and unemployment, and suicidal ideation could not be determined. Second, although item 9 of the Patient Health Questionnaire-9 is widely used to assess suicidal ideation [31,59], a single item may bias the results, and it was not able to explore other domains of suicidality, such as suicidal plans and attempts. Third, online surveys have the inherent bias of self-selection and social desirability, and may lead to greater participation of younger, more educated, and technologically savvy populations. Finally, the assessment of pre-existing sleep problems and mental disorders with a single question was not comprehensive, as some participants were not aware of their diseases, and some may be reluctant to report psychological problems due to stigma.

In conclusion, the prevalence of suicidal ideation in China during the COVID-19 pandemic was relatively high, particularly in cases of COVID-19, frontline workers, individuals with a history of mental health problems, those experiencing quarantine and unemployment, and those with poor health-related information. Provision of health information on infection as well as mental wellness is crucial to reduce the risk of suicidal ideation. These findings contribute to a greater understanding of suicidal behaviors during pandemics, help to identify at-risk populations, and lead to targeted prevention strategies. Longitudinal studies with a broader measurement of suicidal behaviors, as well as systematic evaluation of long-term outcomes are needed.

## Data Availability

We do not make our resources publicly available.

## Supplementary material

For supplementary material accompanying this paper visit https://doi.org/10.1192/j.eurpsy.2021.5. click here to view supplementary material
