# Supplementary material for: Prevalence and correlates of suicidal ideation among the general population in China during the COVID-19 pandemic
Source: Eur Psychiatry. 2021 Feb 3;64(1):e18. doi: 10.1192/j.eurpsy.2021.5 (PMC7943957; doi:10.1192/j.eurpsy.2021.5)
Supplement: Supplementary file 1 [file S0924933821000055sup001.docx]

**Prevalence and Correlates of Suicidal Ideation Among the General Population in China During the COVID-19 Pandemic**

Suicidal ideation during the COVID-19 outbreak in China

**Le Shi,^1, #^ Jian-Yu Que,^1, #^ Zheng-An Lu,^1^ Yi-Miao Gong,^2^ Lin Liu,^3^ Yun-He Wang,^3^ Mao-Sheng Ran,^4^ Nisha Ravindran,^5^ Arun V. Ravindran,^5^ Seena Fazel,^6^ Yan-Ping Bao,^3^ Jie Shi,^3^ Lin Lu,^1, 2, 3,^ ***

^1^Peking University Sixth Hospital, Peking University Institute of Mental Health, NHC Key Laboratory of Mental Health (Peking University), National Clinical Research Center for Mental Disorders (Peking University Sixth Hospital), Beijing, China

^2^Peking-Tsinghua Center for Life Sciences and PKU-IDG/McGovern Institute for Brain Research, Peking University, Beijing, China

^3^National Institute on Drug Dependence and Beijing Key Laboratory on Drug Dependence Research, Peking University, Beijing, China

^4^Department of Social Work and Social Administration, University of Hong Kong, Hong Kong, China

^5^Department of Psychiatry, University of Toronto, Toronto, Canada

^6^Department of Psychiatry, University of Oxford, Oxford, UK

*Corresponding author: Prof. Lin Lu, MD, PhD, Institute of Mental Health and Peking University Sixth Hospital, 51 Huayuanbei Road, Beijing, 100191, China. Tel: +86-10-82805308. Fax: +86-10-62026310. E-mail: [linlu@bjmu.edu.cn](mailto:linlu@bjmu.edu.cn).

**Supplementary Results**

Due to space constraints for the main manuscript, we present additional results here.

**Supplementary Table 1. Demographic characteristics of participants.**

|  | The severity of suicidal ideation | | | | *p* |
| --- | --- | --- | --- | --- | --- |
|  | No | Seldom | Often | Always |  |
| Overall, n(%) | 47,357(83.6) | 6,206(10.9) | 2,320(4.1) | 796(1.4) |  |
| Categorical variables, n(%) |  |  |  |  |  |
| Gender, n(%) |  |  |  |  | <0.001 |
| Male | 21,954(46.4) | 3,418(55.1) | 1,364(58.8) | 413(51.9) |  |
| Female | 25,403(53.6) | 2788(44.9) | 956(41.2) | 383(48.1) |  |
| Age (years), n(%) |  |  |  |  | <0.001 |
| 18-24 | 2,461(5.2) | 495(8.0) | 225(9.7) | 86(10.8) |  |
| 25-34 | 18,747(39.6) | 2,877(46.4) | 1,063(45.8) | 363(45.6) |  |
| 35-44 | 18,477(39.0) | 2,125(34.2) | 788(34.0) | 268(33.7) |  |
| ≥45 | 7,672(16.2) | 709(11.4) | 244(10.5) | 79(9.9) |  |
| Living area, n(%) |  |  |  |  | <0.001 |
| Urban | 44,277(93.5) | 5,722(92.2) | 2,131(91.9) | 709(89.1) |  |
| Rural | 3,080(6.5) | 484(7.8) | 189(8.1) | 87(10.9) |  |
| Geographical region in China, n(%) |  |  |  |  | <0.001 |
| Eastern | 19,441(41.1) | 2,495(40.2) | 947(40.9) | 289(36.4) |  |
| Northern | 8,622(18.2) | 1,088(17.5) | 376(16.2) | 141(17.8) |  |
| Northwest | 1,092(2.3) | 168(2.7) | 64(2.8) | 24(3.0) |  |
| Northeast | 3,234(6.8) | 473(7.6) | 152(6.6) | 62(7.8) |  |
| Central | 3,945(8.3) | 527(8.5) | 243(10.5) | 88(11.1) |  |
| Southern | 8,390(17.7) | 1,094(17.6) | 396(17.1) | 148(18.6) |  |
| Southwest | 2,619(5.5) | 357(5.8) | 138(6.0) | 42(5.3) |  |
| Marital status, n(%) |  |  |  |  | <0.001 |
| Married | 36,964(78.1) | 4,591(74.0) | 1,646(70.9) | 562(70.6) |  |
| Separated/divorced | 1,076(2.3) | 121(1.9) | 55(2.4) | 18(2.3) |  |
| Never married | 9,317(19.7) | 1,494(24.1) | 619(26.7) | 216(27.1) |  |
| Educational level, n(%) |  |  |  |  | <0.001 |
| Middle school and lower | 7,761(16.4) | 1,084(17.5) | 465(20.0) | 230(28.9) |  |
| College and higher | 39,596(83.6) | 5,122(82.5) | 1,855(80.0) | 566(71.1) |  |
| Monthly family income (¥), n(%) |  |  |  |  | <0.001 |
| <5,000 | 10,264(21.7) | 1,752(28.2) | 695(30.0) | 305(38.3) |  |
| 5,000-7,999 | 11,230(23.7) | 1,604(25.8) | 627(27.0) | 202(25.4) |  |
| 8,000-11,999 | 10,851(22.9) | 1,371(22.1) | 471(20.3) | 136(17.1) |  |
| ≥12,000 | 15,012(31.7) | 1,479(23.8) | 527(22.7) | 153(19.2) |  |
| History of mental disorders, n(%) |  |  |  |  | <0.001 |
| No | 47,040(99.3) | 6,080(98.0) | 2,256(97.2) | 761(95.6) |  |
| Unknown | 223(0.5) | 91(1.5) | 43(1.9) | 24(3.0) |  |
| Yes | 94(0.2) | 32(0.6) | 21(0.9) | 11(1.4) |  |
| Family history of mental disorders, n(%) |  |  |  |  | <0.001 |
| No | 46,628(98.5) | 6,040(97.3) | 2,245(96.8) | 762(95.7) |  |
| Unknown | 410(0.9) | 124(2.0) | 49(2.1) | 25(3.1) |  |
| Yes | 319(0.7) | 42(0.7) | 26(1.1) | 9(1.1) |  |
| History of sleep problems, n(%) |  |  |  |  | <0.001 |
| No | 34,825(73.5) | 3,937(63.4) | 1,456(62.8) | 480(60.3) |  |
| Yes | 12,532(26.5) | 2,269(36.6) | 864(37.2) | 316(39.7) |  |
| Have you suspected or confirmed been infected during the COVID-19 pandemic? n(%) | | | | | <0.001 |
| No | 47,320(99.9) | 6,168(99.4) | 2,298(99.1) | 793(99.6) |  |
| Yes | 37(0.1) | 38(0.6) | 22(0.9) | 3(0.4) |  |
| Have you been to the most severely COVID-19 affected region recently? n(%) | | | | | <0.001 |
| No | 45,274(95.6) | 5,886(94.8) | 2,125(91.6) | 697(87.6) |  |
| Yes, I remain at the region | 1,821(3.8) | 281(4.5) | 170(7.3) | 80(10.1) |  |
| Yes, I have left the region | 262(0.6) | 39(0.6) | 25(1.1) | 19(2.4) |  |
| Have you experienced quarantine during the COVID-19 pandemic? n(%) | | | | | <0.001 |
| No | 34,105(72.0) | 4,149(66.9) | 1,478(63.7) | 493(61.9) |  |
| Yes, quarantine duration <14 days | 1,668(3.5) | 349(5.6) | 142(6.1) | 40(5.0) |  |
| Yes, quarantine duration ≥14 days | 11,584(24.5) | 1,708(27.5) | 700(30.2) | 263(33.0) |  |
| Have you experienced the COVID-19 induced unemployment? N(%) | | | | | <0.001 |
| No | 45,933(97.0) | 5,828(93.9) | 2,155(92.9) | 673(84.5) |  |
| Yes | 1,424(3.0) | 378(6.1) | 165(7.1) | 123(15.5) |  |
| Did you participate in frontline work? n(%) | | | | | <0.001 |
| No | 39,495(83.4) | 5,022(80.9) | 1,838(79.2) | 599(75.3) |  |
| Yes | 7,862(16.6) | 1,184(19.1) | 482(20.8) | 197(24.7) |  |
| Have you got more access to psychological knowledge after the COVID-19 pandemic? n(%) | | | | | <0.001 |
| No | 37,822(79.9) | 5,145(82.9) | 1,966(84.7) | 680(85.4) |  |
| Yes | 9,535(20.1) | 1,061(17.1) | 354(15.3) | 116(14.6) |  |
| Continuous variables |  |  |  |  |  |
| The rating of perceived psychological stress after the COVID-19 pandemic [mean (SD)] | 4.06(2.68) | 5.17(2.39) | 5.33(2.52) | 6.32(3.28) | <0.001 |
| The degree of attention to the related information about the COVID-19 pandemic [mean (SD)] | 9.29(1.54) | 8.85(2.03) | 8.74(2.19) | 9.05(2.28) | <0.001 |
| The mastery degree of perceived knowledge of COVID-19 [mean (SD)] | 8.19(1.80) | 7.72(2.10) | 7.63(2.22) | 8.17(2.53) | <0.001 |
| The difficulty in getting access to the information about psychological interventions after the COVID-19 pandemic [mean (SD)] | 3.02(2.46) | 4.34(2.30) | 4.72(2.40) | 5.10(3.29) | <0.001 |

COVID-19, coronavirus disease 2019. SD, standard deviation. The severity of suicidal ideation: Seldom, several days over the last two weeks. Often, more than half the days over the last two weeks. Always, nearly every day over the last two weeks.

**Supplementary Table 2. Factors associated with the various severity of suicidal ideation in the ordinal logistic regression analysis.**

| Variables | OR(95%CI) | *p* |
| --- | --- | --- |
| Gender |  |  |
| Male | 1(ref) |  |
| Female | 0.66(0.63-0.69) | <0.001 |
| Age (years) |  |  |
| 18-24 | 1.94(1.72-2.19) | <0.001 |
| 25-34 | 1.56(1.45-1.70) | <0.001 |
| 35-44 | 1.25(1.15-1.35) | <0.001 |
| ≥45 | 1(ref) |  |
| Living area |  |  |
| Urban | 1(ref) |  |
| Rural | 0.92(0.84-1.01) | 0.077 |
| Geographical region in China |  |  |
| Eastern | 1(ref) |  |
| Northern | 0.94(0.88-1.01) | 0.083 |
| Northwest | 0.96(0.83-1.12) | 0.110 |
| Northeast | 1.04(0.95-1.48) | 0.138 |
| Central | 0.77(0.70-0.85) | <0.001 |
| Southern | 0.83(0.78-0.89) | <0.001 |
| Southwest | 0.91(0.82-1.01) | 0.012 |
| Marital status |  |  |
| Married | 1(ref) |  |
| Separated/divorced | 0.99(0.84-1.16) | 0.873 |
| Never married | 0.93(0.88-1.00) | 0.039 |
| Educational level |  |  |
| Middle school and lower | 1(ref) |  |
| College and higher | 0.92(0.86-0.98) | 0.027 |
| Monthly family income (¥) |  |  |
| <5,000 | 1(ref) |  |
| 5,000-7,999 | 0.86(0.80-0.92) | <0.001 |
| 8,000-11,999 | 0.75(0.70-0.80) | <0.001 |
| ≥12,000 | 0.62(0.58-0.67) | <0.001 |
| History of mental disorders |  |  |
| No | 1(ref) |  |
| Unknown | 1.95(1.51-2.52) | <0.001 |
| Yes | 2.17(1.55-3.04) | <0.001 |
| Family history of mental disorders |  |  |
| No | 1(ref) |  |
| Unknown | 1.12(0.90-1.40) | 0.315 |
| Yes | 0.69(0.52-0.91) | 0.009 |
| History of sleep problems |  |  |
| No | 1(ref) |  |
| Yes | 1.43(1.37-1.51) | <0.001 |
| Have you suspected or confirmed been infected during the COVID-19 pandemic? |  |  |
| No | 1(ref) |  |
| Yes | 2.21(1.48-3.29) | <0.001 |
| Have you been to the most severely COVID-19 affected region recently? |  |  |
| No | 1(ref) |  |
| Yes, I remain at the region | 1.53(1.34-1.73) | <0.001 |
| Yes, I have left the region | 1.69(1.30-2.19) | <0.001 |
| Have you ever experienced quarantine during the COVID-19 pandemic? |  |  |
| No | 1(ref) |  |
| Yes, quarantine duration <14 days | 1.35(1.21-1.50) | <0.001 |
| Yes, quarantine duration ≥14 days | 1.11(1.05-1.17) | <0.001 |
| Have you experienced the COVID-19 induced unemployment? |  |  |
| No | 1(ref) |  |
| Yes | 1.82(1.64-2.01) | <0.001 |
| Did you participate in frontline work? |  |  |
| No | 1(ref) |  |
| Yes | 1.22(1.15-1.30) | <0.001 |
| Have you got more access to psychological knowledge after the COVID-19 pandemic? |  |  |
| No | 1(ref) |  |
| Yes | 0.73(0.69-0.78) | <0.001 |
| The rating of perceived psychological stress after the COVID-19 pandemic | 1.13(1.12-1.14) | <0.001 |
| The degree of attention to the related information about COVID-19 pandemic | 0.88(0.87-0.89) | <0.001 |
| The mastery degree of perceived knowledge of COVID-19 | 0.96(0.95-0.97) | <0.001 |
| The difficulty in getting access to the information about psychological interventions after the COVID-19 pandemic | 1.19(1.18-1.20) | <0.001 |
| Nagelkerke R^2^ | 0.134 | |

**Supplementary Table 3. Factors that were associated with suicidal ideation among different groups of changes in psychological stress after the COVID-19 pandemic.**

| Variables | Changes in psychological stress after the COVID-19 pandemic | | |
| --- | --- | --- | --- |
|  | Increased | No change | Decreased |
| Gender |  |  |  |
| Male | 1(ref) | 1(ref) | 1(ref) |
| Female | 0.68(0.63-0.74)^***^ | 0.70(0.64-0.76)^***^ | 0.59(0.54-0.65)^***^ |
| Age (years) |  |  |  |
| 18-24 | 1.84(1.52-2.24)^***^ | 1.97(1.58-2.44)^***^ | 2.02(1.59-2.57)^***^ |
| 25-34 | 1.63(1.43-1.85)^***^ | 1.59(1.38-1.83)^***^ | 1.43(1.22-1.67)^***^ |
| 35-44 | 1.31(1.15-1.48)^***^ | 1.32(1.16-1.52)^***^ | 1.05(0.90-1.22) |
| ≥45 | 1(ref) | 1(ref) | 1(ref) |
| Living area |  |  |  |
| Urban | 1(ref) | 1(ref) | 1(ref) |
| Rural | 0.98(0.85-1.13) | 0.92(0.78-1.07) | 0.90(0.75-1.08) |
| Geographical region in China |  |  |  |
| Eastern | 1(ref) | 1(ref) | 1(ref) |
| Northern | 0.91(0.82-1.01) | 1.00(0.89-1.12) | 0.98(0.86-1.13) |
| Northwest | 0.89(0.70-1.13) | 1.03(0.88-1.21) | 1.08(0.81-1.44) |
| Northeast | 1.09(0.94-1.28) | 0.96(0.74-1.25) | 1.07(0.89-1.30) |
| Central | 0.79(0.67-0.93)^**^ | 0.82(0.68-0.99)^*^ | 0.73(0.59-0.89)^**^ |
| Southern | 0.85(0.76-0.94)^**^ | 0.91(0.81-1.02) | 0.77(0.67-0.89)^***^ |
| Southwest | 0.86(0.73-1.02) | 1.00(0.83-1.19) | 0.90(0.72-1.11) |
| Marital status |  |  |  |
| Married | 1(ref) | 1(ref) | 1(ref) |
| Separated/divorced | 1.09(0.84-1.40) | 1.04(0.79-1.37) | 0.72(0.50-1.03) |
| Never married | 1.01(0.91-1.11) | 0.94(0.84-1.05) | 0.77(0.68-0.89)^***^ |
| Educational level |  |  |  |
| Middle school and lower | 1(ref) | 1(ref) | 1(ref) |
| College and higher | 0.96(0.86-1.07) | 0.89(0.79-0.99)^*^ | 1.03(0.90-1.17) |
| Monthly family income (¥) |  |  |  |
| <5,000 | 1(ref) | 1(ref) | 1(ref) |
| 5,000-7,999 | 0.81(0.73-0.90)^***^ | 0.95(0.85-1.06) | 0.86(0.75-0.98)^*^ |
| 8,000-11,999 | 0.77(0.69-0.86)^***^ | 0.81(0.72-0.91)^**^ | 0.74(0.64-0.85)^***^ |
| ≥12,000 | 0.63(0.56-0.70)^***^ | 0.65(0.57-0.73)^***^ | 0.65(0.57-0.75)^***^ |
| History of mental disorders |  |  |  |
| No | 1(ref) | 1(ref) | 1(ref) |
| Unknown | 2.25(1.48-3.42)^***^ | 1.45(0.91-2.29) | 2.32(1.31-4.11)^**^ |
| Yes | 2.53(1.36-4.73)^**^ | 1.63(0.91-2.92) | 2.51(1.19-5.29)^*^ |
| Family history of mental disorders |  |  |  |
| No | 1(ref) | 1(ref) | 1(ref) |
| Unknown | 1.33(0.95-1.86) | 1.45(0.91-2.29) | 1.06(0.63-1.79) |
| Yes | 0.66(0.42-1.03) | 1.63(0.91-2.92) | 0.64(0.35-1.18) |
| History of sleep problems |  |  |  |
| No | 1(ref) | 1(ref) | 1(ref) |
| Yes | 1.44(1.34-1.56)^***^ | 1.77(1.63-1.93)^***^ | 1.37(1.24-1.51)^***^ |
| Have you suspected or confirmed been infected during the COVID-19 pandemic? |  |  |  |
| No | 1(ref) | 1(ref) | 1(ref) |
| Yes | 4.82(2.05-11.32)^***^ | 4.22(1.97-9.02)^***^ | 2.03(0.85-4.84) |
| Have you been to the most severely COVID-19 affected region recently? |  |  |  |
| No | 1(ref) | 1(ref) | 1(ref) |
| Yes, I remain at the region | 1.34(1.09-1.66)^**^ | 1.55(1.23-1.94)^***^ | 1.83(1.42-2.36)^***^ |
| Yes, I have left the region | 1.60(1.06-2.42)^*^ | 1.29(0.73-2.27) | 1.56(0.97-2.50) |
| Have you ever experienced quarantine during the COVID-19 pandemic? |  |  |  |
| No | 1(ref) | 1(ref) | 1(ref) |
| Yes, quarantine duration <14 days | 1.35(1.14-1.60)^**^ | 1.46(1.21-1.77)^***^ | 1.42(1.15-1.77)^***^ |
| Yes, quarantine duration ≥14 days | 1.12(1.03-1.22)^*^ | 1.13(1.03-1.24)^**^ | 1.11(1.00-1.24)^*^ |
| Have you experienced the COVID-19 induced unemployment? |  |  |  |
| No | 1(ref) | 1(ref) | 1(ref) |
| Yes | 2.12(1.80-2.49)^***^ | 1.66(1.39-1.98)^***^ | 1.64(1.33-2.03)^***^ |
| Did you participate in frontline work? |  |  |  |
| No | 1(ref) | 1(ref) | 1(ref) |
| Yes | 1.21(1.10-1.33)^***^ | 1.21(1.09-1.34)^***^ | 1.22(1.08-1.37)^**^ |
| Have you got more access to psychological knowledge after the COVID-19 pandemic? |  |  |  |
| No | 1(ref) | 1(ref) | 1(ref) |
| Yes | 0.79(0.72-0.86)^***^ | 0.91(0.81-1.02) | 0.78(0.68-90)^**^ |
| The degree of attention to the related information about COVID-19 pandemic | 0.88(0.86-0.90)^***^ | 0.90(0.88-0.92)^***^ | 0.90(0.87-0.93)^***^ |
| The mastery degree of perceived knowledge of COVID-19 | 0.96(0.93-0.98)^***^ | 0.97(0.94-0.99)^**^ | 0.95(0.92-0.97)^***^ |
| The difficulty in getting access to the information about psychological interventions after the COVID-19 pandemic | 1.19(1.18-1.21)^***^ | 1.30(1.28-1.32)^***^ | 1.31(1.28-1.34)^***^ |
| Nagelkerke R^2^ | 0.109 | 0.170 | 0.166 |

^*^*p*<0.05, ^**^*p*<0.01, ^***^*p*<0.001
